# Olecranon flip osteotomy for distal humerus fractures

**DOI:** 10.1007/s00064-026-00957-6

**Published:** 2026-09-15

**Authors:** Nicole Maria van Veelen, Klaas Victor, Simon Lambert, Frank J. P. Beeres, Reto Babst

**Affiliations:** 1 Department of Orthopaedic and Trauma Surgery, Luzerner Kantonsspital, Spitalstrasse, 6000 Luzern 16, Switzerland; 2 Faculty of Health Sciences and Medicine, University of Lucerne, Alpenquai 4, 6005 Lucerne, Switzerland; 3 The Princess Grace Hospital, 30 Devonshire Street, W1G 6PU London, UK

**Keywords:** Distal humerus, Elbow, Anconeus, Osteosynthesis, Triceps, Distaler Humerus, Ellenbogen, Anconeus, Trizeps, Osteosynthese

## Abstract

**Objective:**

Present the olecranon flip osteotomy approach for the treatment of distal humerus fractures as an alternative to the chevron osteotomy or previously described triceps-sparing approaches.

**Indications:**

Distal humerus fractures requiring direct visualization of the articular surface.

**Contraindications:**

Patient unfit for surgery.

**Surgical technique:**

A longitudinal skin incision starting 10 cm proximal to the olecranon tip is performed. The incision curves radially around the tip of the olecranon and continues distally, parallel to the ulnar crest. Identify and protect the ulnar nerve. Proximally a plane along the ulnar edge of the triceps muscle and tendon is developed, releasing the long head of the triceps from the medial intermuscular septum to gain access to the distal humerus. Distally the facia is dissected off the ulna. A thin chip of bone is elevated from the tip of the olecranon together with the triceps tendon and fascia allowing it to be flipped to the radial side. After dissecting the capsule and triceps off the humerus, the distal humerus can be visualized.

**Postoperative management:**

Immediate functional nonweight-bearing rehabilitation with no limitations regarding range of motion. Sling or splint for comfort during the first few days. Graduated weight-bearing after 6 weeks.

**Results:**

The authors have successfully applied the flip osteotomy in 12 cases, one of which was treated with a hemi-arthroplasty. All osteosyntheses achieved fracture healing and all patients acquired good elbow function after 3–6 months.

## Abstract (de)

**Vorbemerkungen:**

Darstellung des Olecranon-Flip-Zugangs für die Behandlung distaler Humerusfrakturen als Alternative zu Chevron-Osteotomien oder zuvor beschriebenen Trizeps-schonenden Zugängen.

**Indikationen:**

Distale Humerusfrakturen, welche Einsicht auf die humerale Gelenkfläche erfordern.

**Kontraindikationen:**

Fehlende Operationsfähigkeit.

**Operationstechnik:**

Setzen einer longitudinalen Hautinzision, die rund 10 cm proximal der Olecranonspitze beginnt, die Olecranonspitze radial umschneidet und distal, parallel zur Ulnakante weitergeführt wird. Der N. ulnaris wird identifiziert und geschützt. Etablieren einer Schicht entlang der ulnaren Begrenzung der Trizepsmuskulatur respektive -sehne, wobei der lange Kopf des Trizeps vom medialen Septum intermusculare gelöst wird, um Zugang zum distalen Humerus zu erhalten. Distal wird die Faszie von der Ulna abpräpariert. Eine dünne Knochenschuppe wird zusammen mit der Trizepssehne und der ulnaren Faszie von der Olecranonspitze abgehoben, sodass sie dann zur radialen Seite weggeklappt werden kann. Anschließend werden die Gelenkkapsel und der Trizepsmuskel vom Humerus gelöst, um Einsicht auf den distalen Humerus und der Gelenkfläche zu erhalten.

**Weiterbehandlung:**

Eine funktionelle Nachbehandlung mit belastungsfreier Mobilisation ohne Einschränkung des Bewegungsumfanges ist direkt postoperativ erlaubt. Eine Schlinge oder Schiene kann in den ersten Tagen zum Komfort getragen werden. Belastungssteigerung ab der 7. postoperativen Woche.

**Ergebnisse:**

Die Flip-Osteotomie wurde in 12 Fällen erfolgreich von den Autoren angewendet. Einer dieser Fälle erhielt eine Hemi-Arthroplastik. Die Frakturheilung konnte in allen Osteosynthesen erreicht werden, und sämtliche Patienten wiesen nach 3–6 Monaten eine gute Ellenbogenfunktion auf.

## Introductory remarks

While distal humerus fractures can occur in all age groups, they are most frequent in elderly women, due to the prevalence of osteoporosis in that population [4]. Distal humerus fractures are uncommon with an annual occurrence of 10–13/10,000, comprising 1–2% of all adult fractures [7]. These fractures remain challenging to treat due to the complex anatomy of the elbow, and the challenge of sufficient surgical exposure. The majority of distal humerus fractures are treated with open reduction and internal fixation (ORIF). In some cases, a total or hemi-prothesis may be the better option (severely comminuted fractures, very distal fractures with coronal shear or fractures with pre-existing osteoarthritis). Nonsurgical management is reserved for very low-demand patients or those that have strict contraindications for an operation.

Extra-articular or simple intra-articular distal humerus fractures can be fixed with limited or no visualization of the joint, either using a posterior paratricipital approach or posterior triceps-split [1]. For coronal shearing injuries, in which the posterior column is intact, an anterior or a lateral approach can be used [9]. For more complex intra-articular fractures joint visualization is required to be able to achieve anatomical reduction of the articular surface. This is very often achieved through a posterior approach with a distal apex chevron osteotomy of the olecranon, which provides more exposure of the articular surface than alternatives such as the triceps-split and paratricipital approaches (52% versus 37% and 26%, respectively) [5]. While the chevron osteotomy permits a very good view of the joint, it also bears the risk of complications. Nonunion of the osteotomy has been reported to occur in 2–4% [2, 13] and implant removal is necessary in over a third of all patients [10]. Further potential complications include malunion, surgical site infections, and implant failure. An important disadvantage of the chevron osteotomy is that the anconeus muscle, the posterolateral stabilizer of the elbow, can be denervated since it receives innervation from a specific branch of the radial nerve which passes through the medial head of the triceps into the anconeus [11].

To avoid the chevron osteotomy and its potential complications alternative “triceps-on” approaches have been described. Zumstein et al. described an extra-articular osteotomy with an anconeus flap [15]. With this approach the extensor mechanism is disrupted as the osteotomy is reflected proximally. At the end of the procedure the osteotomy must be fixed with a cerclage wire technique or screws, with similar risks of complications as the chevron osteotomy. O’Driscoll et al. described the triceps reflecting anconeus pedicle (TRAP) approach [11]. The triceps is mobilized and released together with the periosteum of the olecranon from the medial side (as described previously by Bryan and Morrey [3]); the anconeus is mobilized subperiosteally and remains attached to the triceps as it is mobilized from the lateral side. The entire extensor mechanism can then be reflected off the olecranon to gain exposure of the joint. The tendon–periosteal flap is sutured back in place at the end of the procedure. The percentage of articular surface that is exposed remains smaller than when the chevron osteotomy is used (52% versus 46%) [14]. Habib et al. described a similar approach involving an extra-articular osteotomy of the olecranon, in which a thick wafer of bone is created at the triceps insertion site. This wafer is then reflected together with the triceps and anconeus pedicle. At the conclusion of the procedure, the wafer is reattached by drilling holes in both the wafer and the ulna, allowing it to be securely sutured back into place [6].

In this paper, the authors describe an alternative in which a thin medallion of the olecranon tip is elevated together with the extensor mechanism and everted or ‘flipped’ to the radial side, while preserving the anconeus in continuity with the medial head of the triceps or to the radial side with the entire triceps in continuity with the anconeus, similar in principle to the bigastric trochanter osteotomy for exposure of the hip joint [12]. In contrast to the TRAP or the Bryan Murrey approach, this technique permits the triceps complex to heal via the bone chip, allowing more reliable bone-to-bone healing rather than tendon-to-bone healing.

## Surgical principles and objective

The aim when performing an osteosynthesis of intra-articular distal humerus fractures is to acquire anatomical reduction and sufficient stability to allow functional aftercare. When choosing the approach, the fracture morphology, individual needs of the patient, and potential complications are considered. The flip osteotomy provides good joint exposure (comparable to the TRAP approach) for fracture care and allows the surgeon to preserve the anconeus muscle in continuity with the entire triceps when the flip is turned to the radial side.

## Advantages

- Extensor mechanism of the elbow remains intact
- Olecranon joint remains intact
- Anconeus sparing
- Better visualization of distal humerus than paratricipital approach (equal to TRAP)
- Bone-to-bone healing of the extensor apparatus
- No requirement for plate or tension band wire fixation of osteotomy and therefore less soft-tissue irritation

## Disadvantages

Visualization of ventral trochlea is limited.

## Indications

Intra-articular distal humerus fractures are generally an indication for open reduction and internal fixation (ORIF). In some selected cases with severe comminution or in elderly patients a fracture prosthesis (hemi or full arthroplasty) may be the better option and in very low demand patients or those with strict contraindications for any surgical procedure, nonsurgical treatment may be the only option [9]. The choice of approach used for the ORIF depends on fracture morphology, as each approach allows for a different region and extent of the joint to become visible [2]. Based on this we propose the following indications:

- AO 13 C1 and C2 fractures requiring direct visualization of the join surface
- Fractures in which an intraoperative change of tactics from osteosynthesis to joint replacement may be required

## Contraindications

- Patient unfit for surgery

## Patient information

- General surgical risks
- Implant-related complications such as loss of reduction, screw perforation in ORIF
- Implant related complications and weight-bearing restrictions in elbow arthroplasty (EA)
- Injury to neighboring structures such as nerves and blood vessels (especially ulnar nerve)
- Potential need for implant removal
- Loss of full range of motion and strength
- Posttraumatic osteoarthritis (when osteosynthesis is performed)

## Preoperative work-up

- Clinical examination documenting motor and sensory function of the nerves, especially the ulnar nerve
- Conventional x‑rays in two orthogonal planes (anteroposterior and lateral views)
- A computed tomography (CT) is highly recommended, if possible, with three-dimensional (3D) reconstructions. These facilitate fracture assessment and planning of the osteosynthesis or joint replacement and surgical approach.
- In patients with compromised soft-tissues or polytraumatized patients, it can be beneficial to initially perform closed reduction and temporary stabilization with a joint-bridging external fixator. In such cases the CT should be performed after application of the external fixator as it is easier to understand the fracture and plan the definitive osteosynthesis when the fracture is partially reduced by means of traction (span–scan–plan).
- Single-shot antibiotic prophylaxis should be administered prior to skin incision.

## Instruments

- C‑arm
- Standard surgical instruments for osteosynthesis or joint replacement
- Angular stable plates for distal humerus
- Fine osteotomes
- Large cannula
- Sterile tourniquet (optional)

## Anaesthesia and positioning

**Fig. 1:**
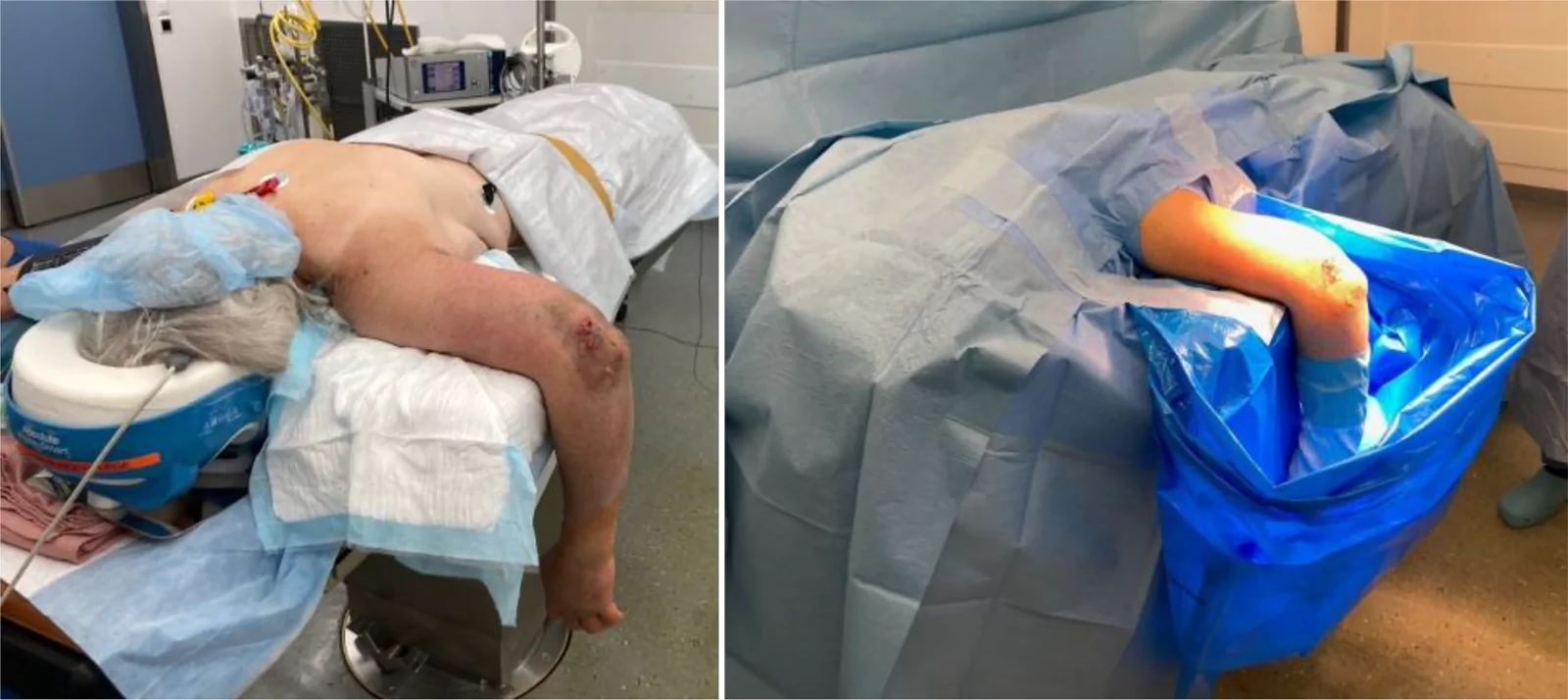
The patient is positioned prone with the arm resting on a short arm bolster

- Prone or lateral decubitus position with the upper arm supported by an arm roll or short arm table (Fig. 1) allowing the elbow to spontaneously flex 90°.
- Due to the positioning general anaesthesia is recommended.
- The C‑arm is positioned by the head of the patient.
- A sterile tourniquet can be placed after washing and draping.

## Surgical technique

(Figs. 2, 3, 4, 5, 6, 7, 8 and 9).

**Fig. 2:**
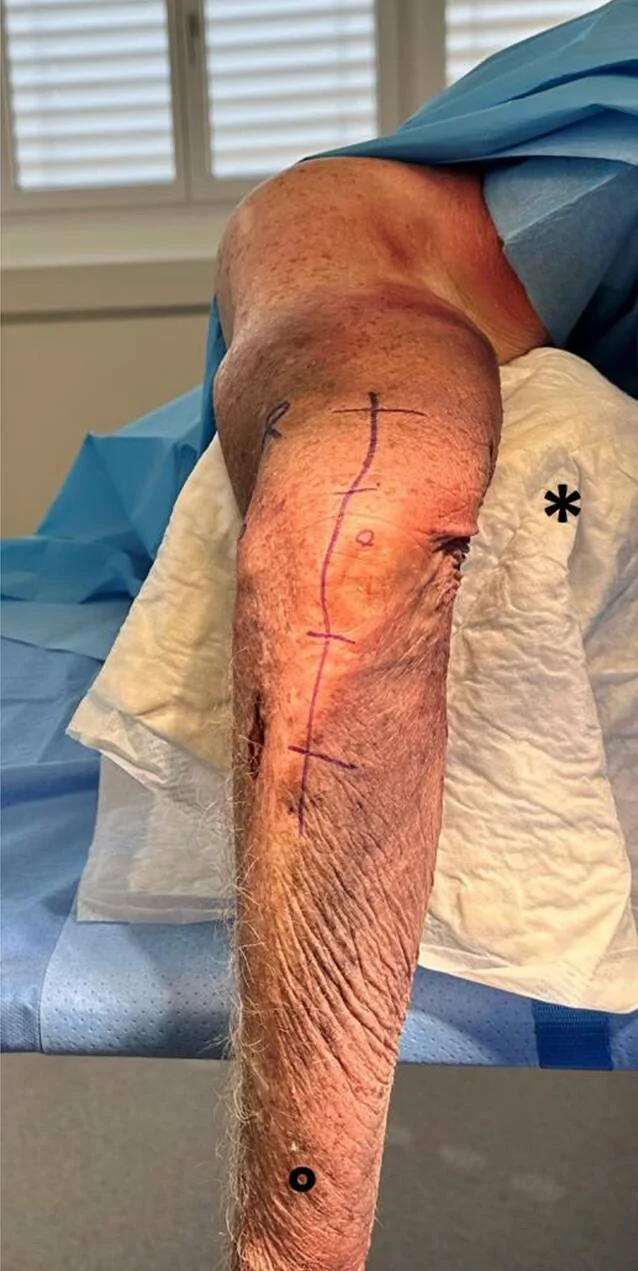
The patient is positioned either in a lateral decubital position or prone. The arm is abducted 90° and placed on a short bolster allowing the elbow to spontaneously flex and the forearm to hang. * Indicates the ulnar side of the elbow, ° indicates distal. A longitudinal skin incision starting approximately 10 cm proximal to the olecranon tip is performed. The incision curves radially around the tip of the olecranon and continues distally, parallel to the ulnar crest

**Fig. 3:**
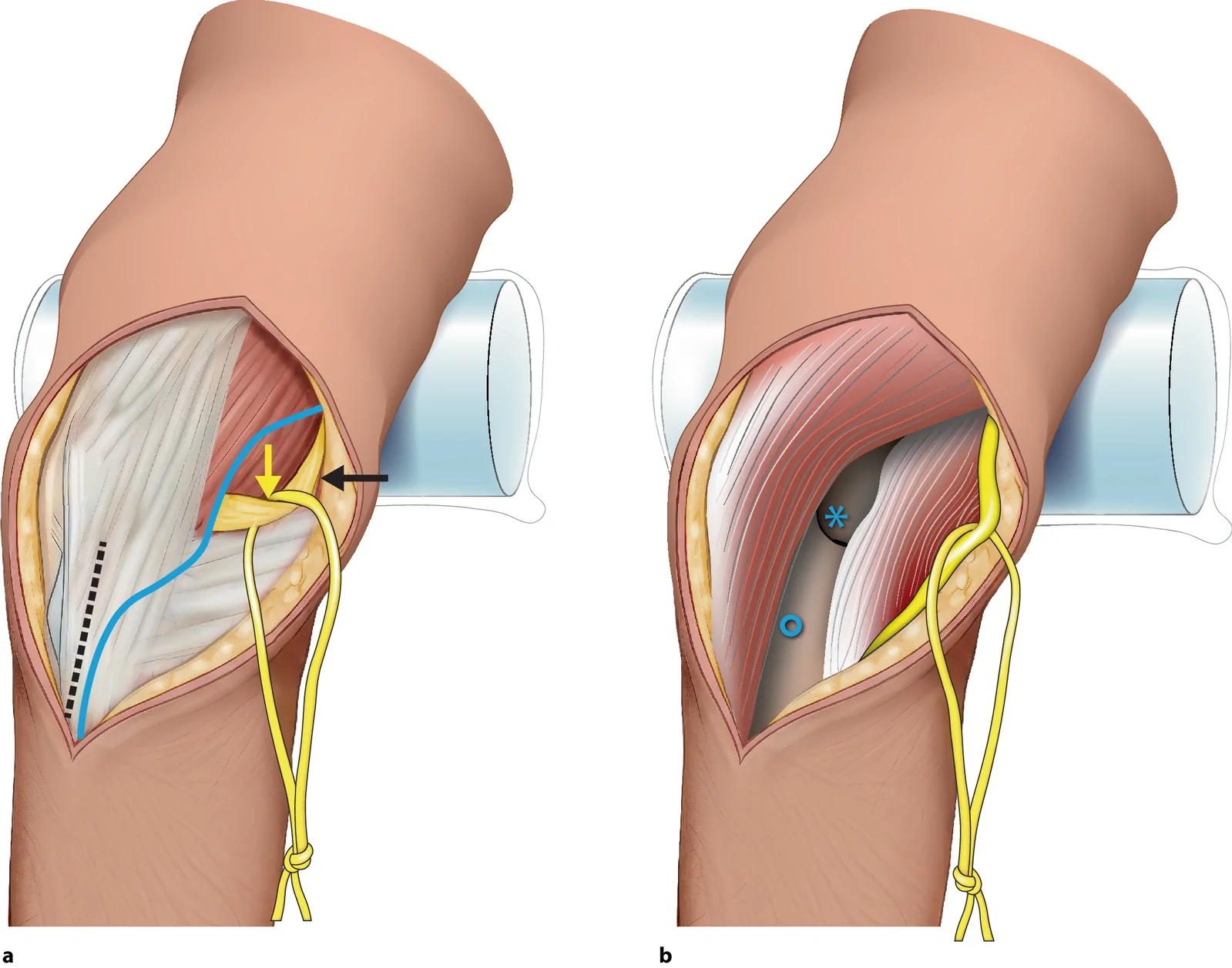
After incising the subcutaneous tissue in line with the skin incision the ulnar nerve is identified, dissected until the first motor branch (often the only motor branch to flexor carpi ulnaris, which therefore should be protected) and marked with a loop. The black arrow marks the ulnar nerve, the yellow arrow marks the first motor branch. The fascia is incised 5 mm ulnar to the olecranon tip and the crest of the ulna. This maintains the integrity of the extensor muscles and allows for refixation of the fascia at the end of the procedure. Proximally, a plane along the ulnar edge of the triceps muscle and tendon is developed, releasing the long head of the triceps from the medial intermuscular septum to gain access to the distal humerus. **a** The ulnar crest is marked with a black dashed line. The planned incision is marked with a blue line. **b** After the fascia is incised the distal humerus and proximal ulna can be seen. *distal humerus, ° proximal ulna

**Fig. 4:**
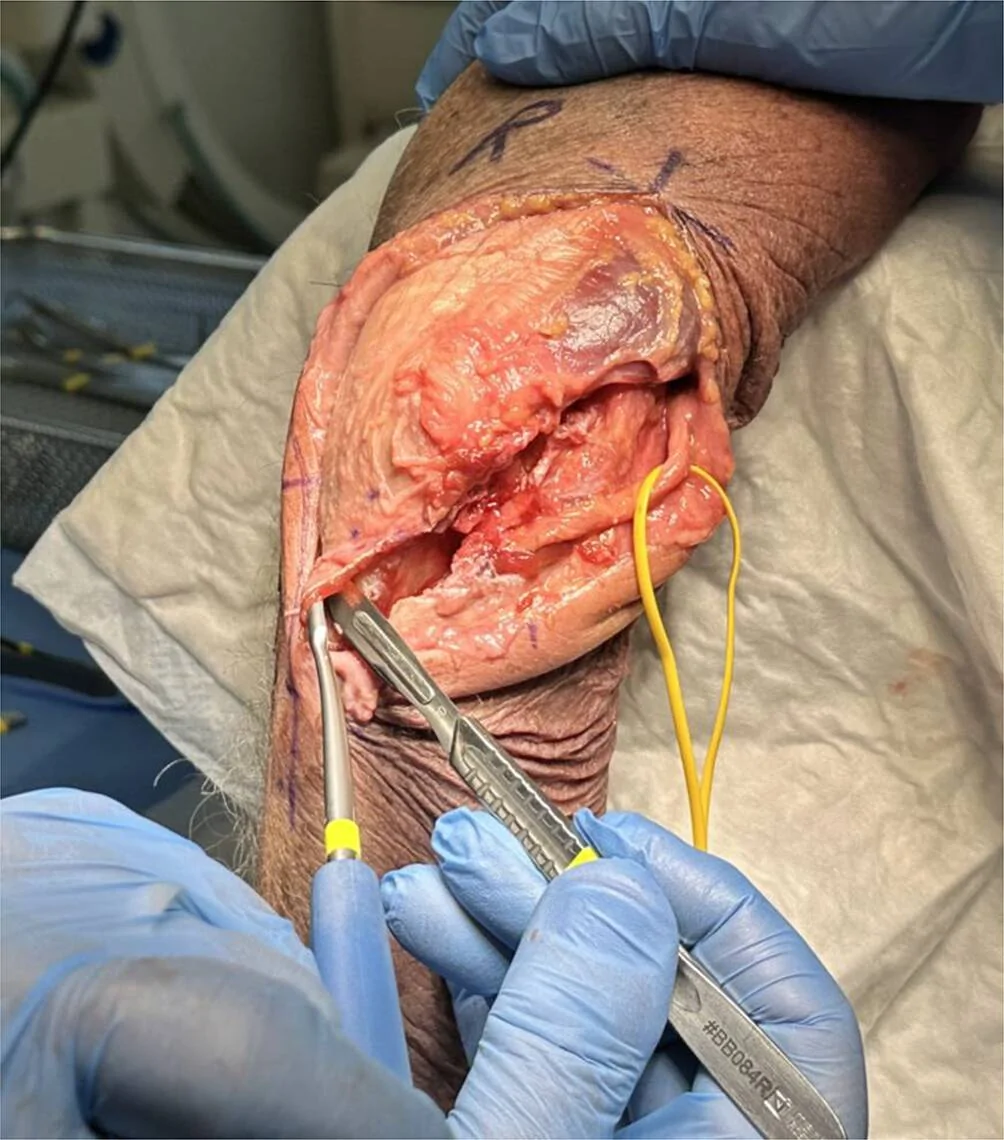
Dissect the antebrachial fascia off the ulna distal to the olecranon, remaining in the periosteal layer. Continue this dissection approximately 5 cm distal to the tip of the olecranon, to avoid tearing of the fascia when the olecranon is flipped. At the tip of the olecranon only release enough of the fascia/tendon to see where the osteotomy will be performed

**Fig. 5:**
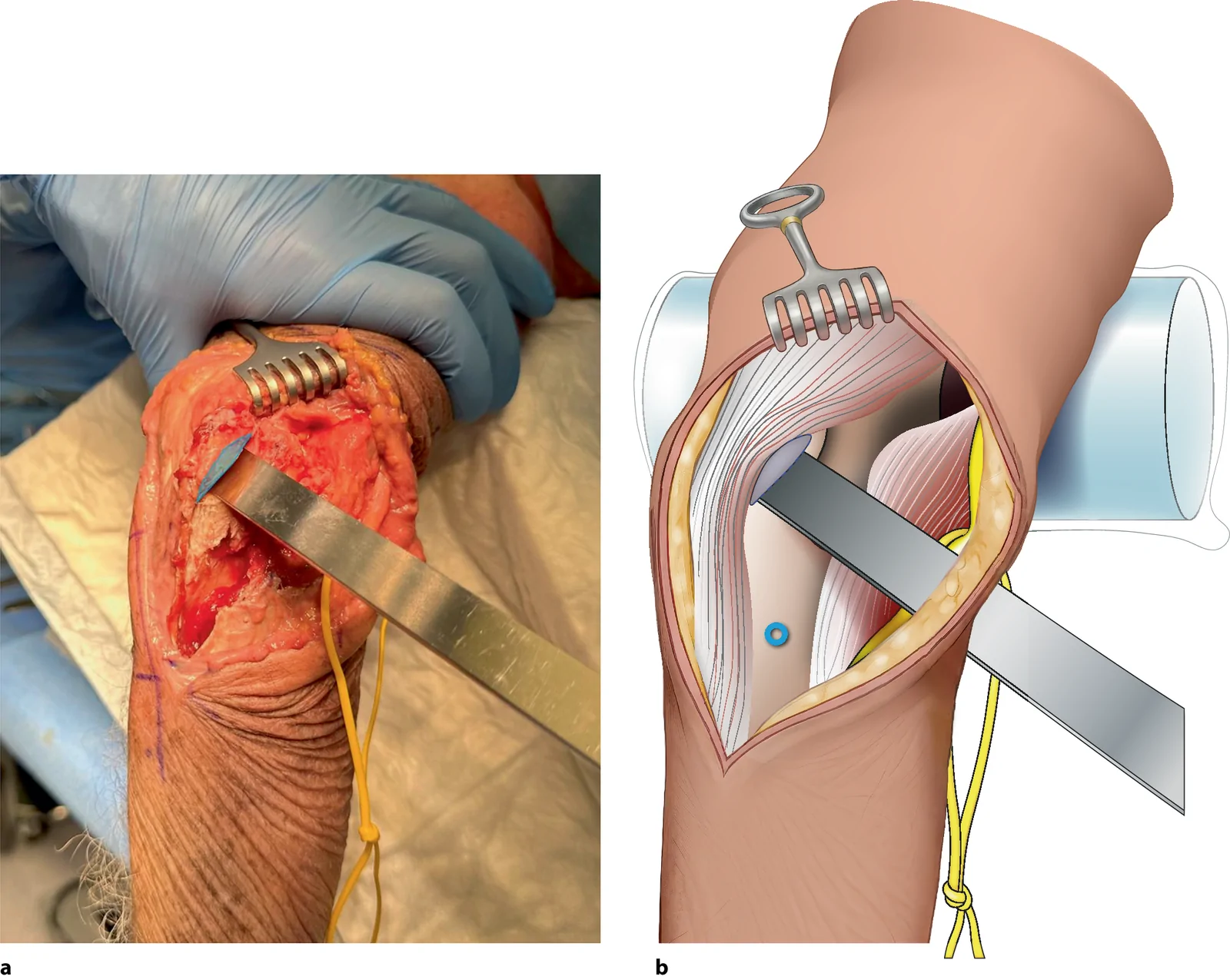
**a,** **b** Using an osteotome a thin chip of bone is elevated from the dorsal aspect of the olecranon together with the intact triceps tendon proximally and antebrachial fascia distally. The bone chip is marked blue. The hook is holding the triceps muscle

**Fig. 6:**
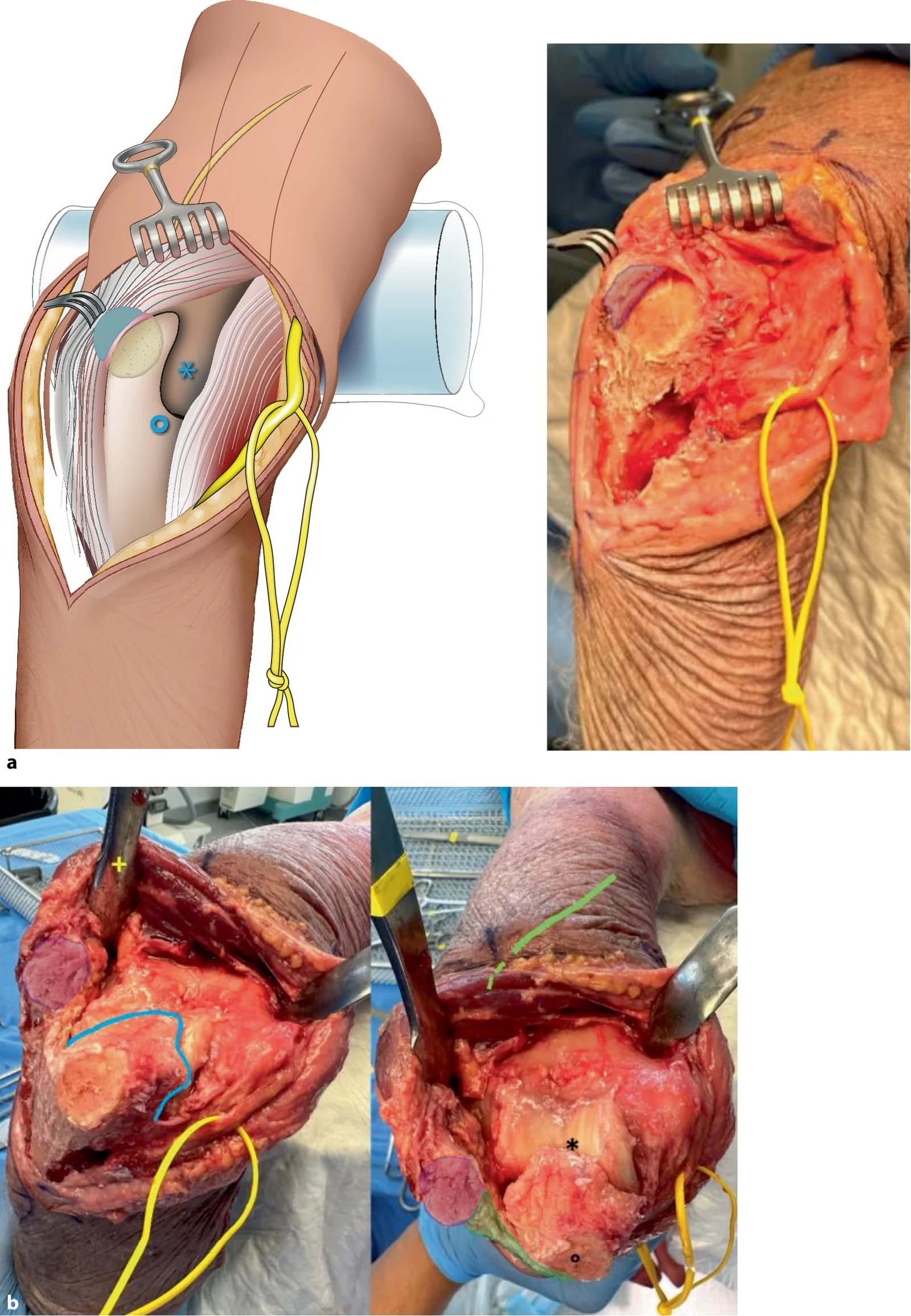
The bone chip can then be reflected toward the radial side together with the intact triceps tendon and antebrachial facia. The capsule and deep aspect of the triceps are then dissected off the humerus allowing for a good view onto the distal humerus. **a** The osteotomy has been completed. The bone chip is marked blue. The ulna is marked with °. The capsule and triceps are still attached to the humerus (*) proximal to the bone chip. **b** After dissecting the medial head of the triceps off the distal humerus below the spiral groove (beware of the radial nerve) a Hohman retractor (+) can be placed on the radial side of the humerus. The bone chip is marked purple. The margin of the olecranon is marked with a blue line. **c** After dissecting the capsule a clear view onto the distal humerus is achieved. By flexing the elbow, the joint surface can be seen. The intact antebrachial fascial continuation of the triceps tendon is marked in green, * marks the distal humerus, ° marks the olecranon. The expected location of the radial nerve has been marked in green. The solid line represents the portion of the nerve on the dorsal aspect of the humerus in the spiral groove, the dashed line is where it is expected to pierce the intramuscular septum

**Fig. 7:**
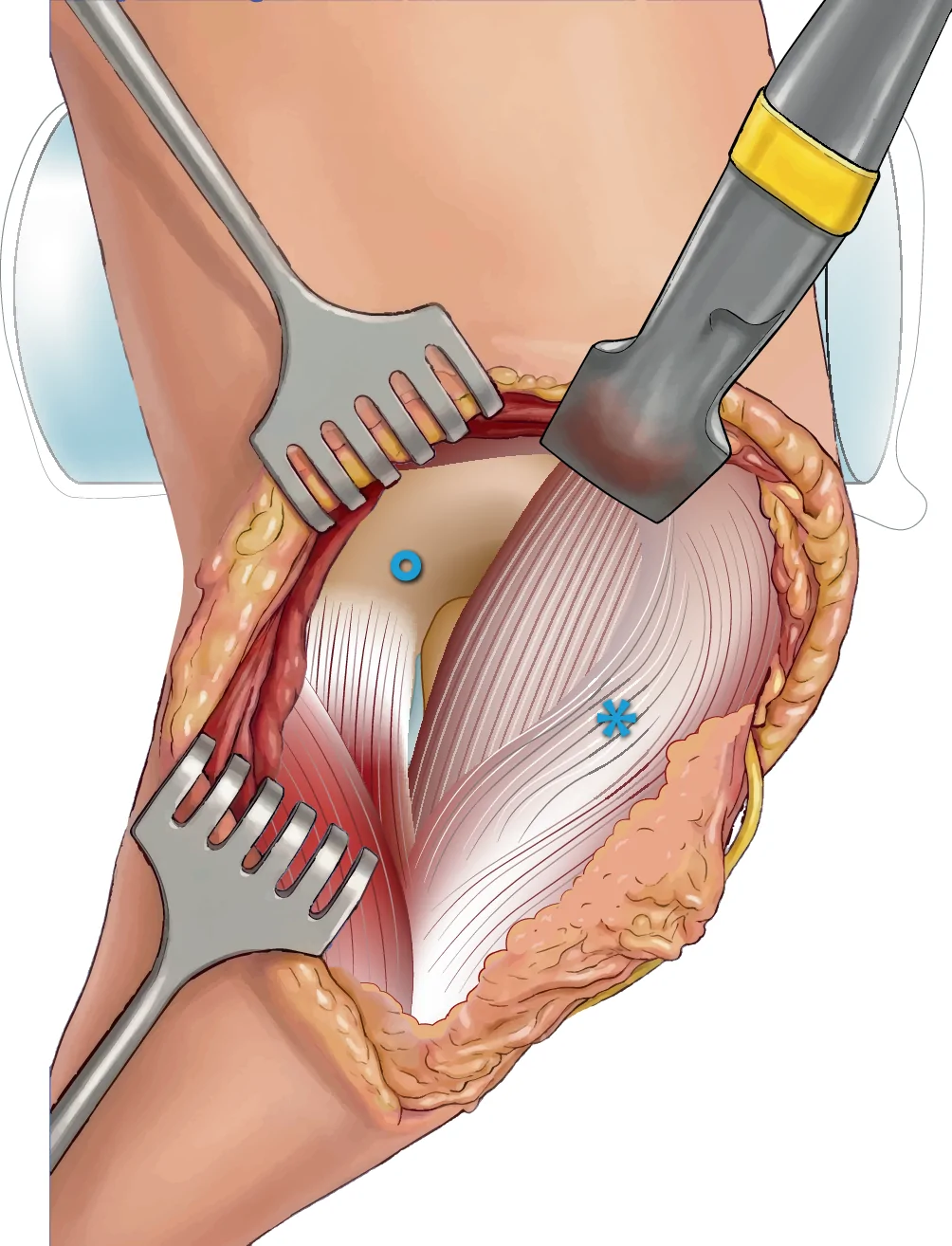
To gain a better view of the radial supracondylar ridge of the humerus the triceps can be dissected off the humerus radially, as would be done in a paratriceps approach. * marks the triceps which is reflected toward the ulna to gain visibility of the radial aspect of the humerus (°). The bone chip cannot be seen as it has been laid back onto the olecranon and is covered by the intact extensor mechanism

**Fig. 8:**
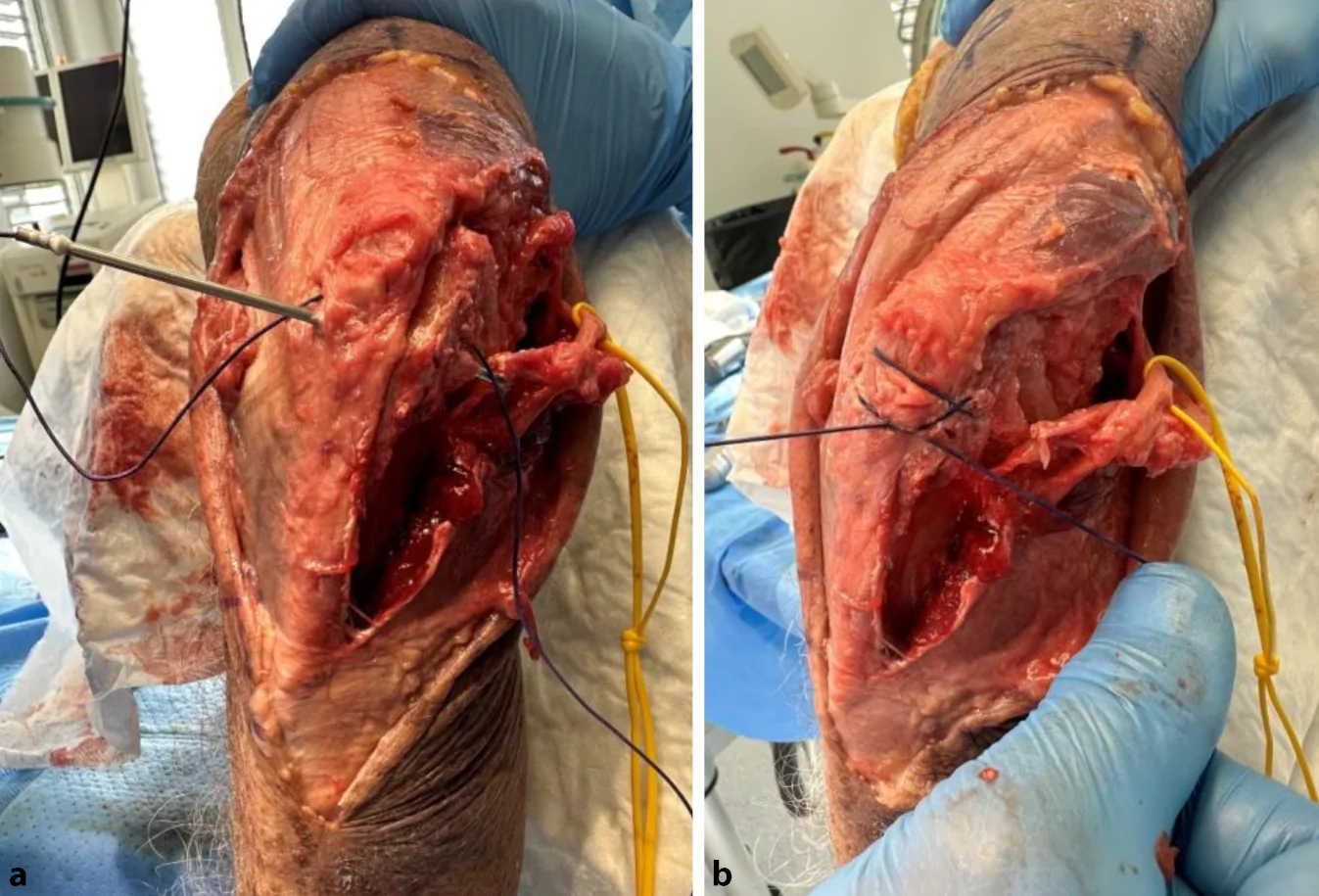
After the osteosynthesis has been completed, the bone chip can be flipped back to lay on the olecranon. It is refixed with a transosseous suture. The authors did this by drilling two parallel holes in the olecranon and passing the suture through the holes with the help of a large cannula. A figure-of-eight suture was then performed. **a** The bone chip has been relocated. The transosseous sutures for refixation are placed with the help of a large cannula. **b** The figure-of-eight suture secures the bone chip back onto the olecranon. The intact extensor mechanism is visible

**Fig. 9:**
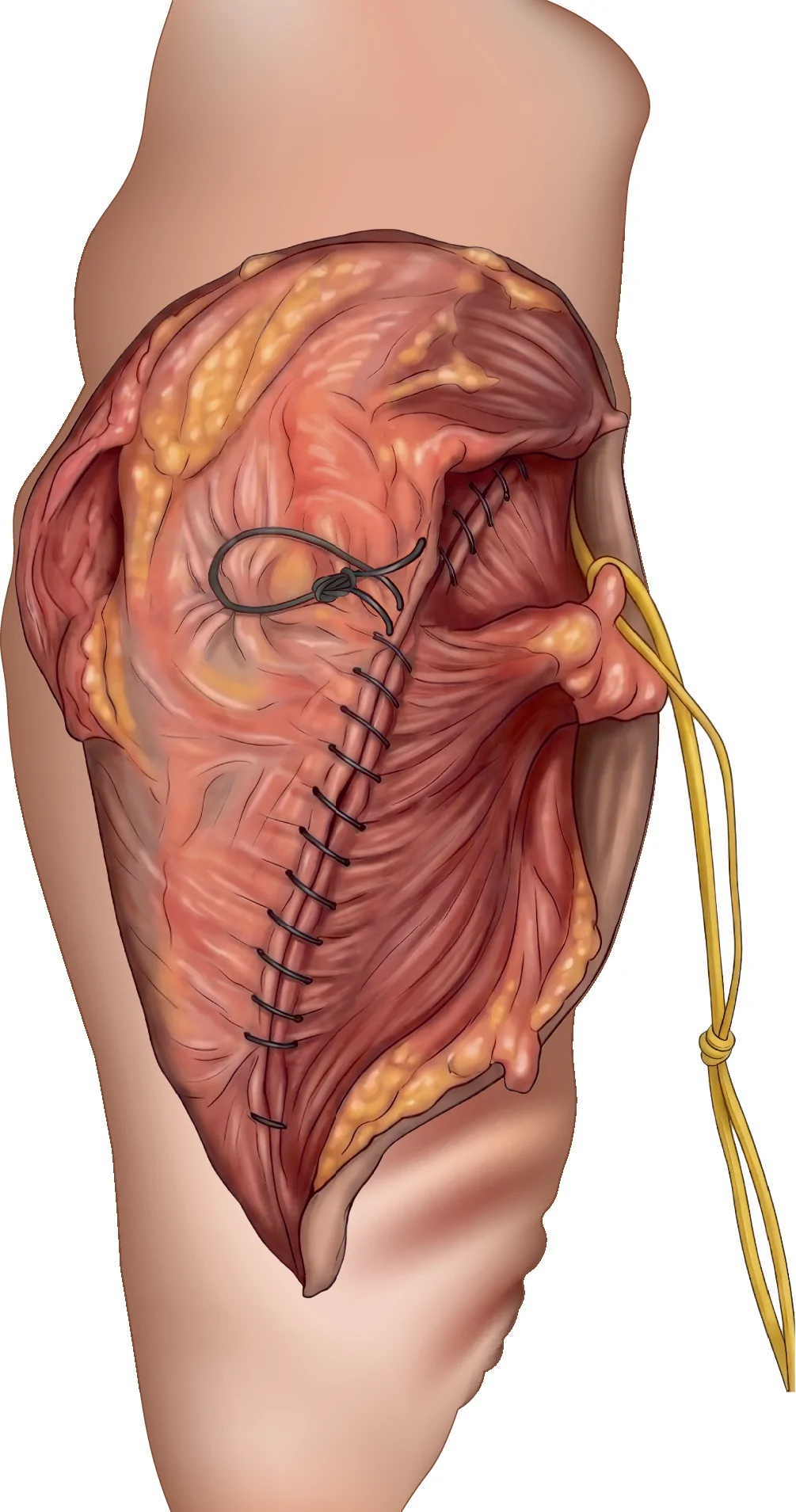
After securing the bone chip, the antebrachial fascia is sutured prior to closing the incision. The ulnar nerve is not transposed unless the implant position or fracture morphology is likely to cause irritation

## Postoperative management

- Postoperative x‑ray in two planes to check adequate reduction and correct implant positioning if no sufficient intraoperative images were obtained
- Sling or splint for comfort and to allow soft tissue healing
- Immediate nonweight-bearing range of motion exercises including sagittal plane bending within the limits of discomfort on a table surface (to reduce the effect of gravity) and isometric common flexor and extensor muscle strengthening under the guidance of a physical therapist
- Clinical and radiological follow-up 6 weeks postoperatively, after which isokinetic (resistance) training and graduated weight-bearing can commence, according to the completeness of fracture healing
- The duration of rehabilitation until the patient can return to work depends greatly on the occupation. In the case of a desk job, it can be expected that the patient can return after soft tissue healing has been achieved, approximately 2 weeks postoperatively. For physically strenuous jobs, return to work will likely take at least 3 months.

## Errors, hazards, complications

- The ulnar nerve (and to a lesser extent also the radial nerve) are exposed and at risk of injury throughout the operation
- Fracture fixation errors such as screw perforation into the elbow joint or olecranon fossa
- Failure of healing/secondary rupture or avulsion of the osteotomy or triceps
- Decreased range of motion

## Results

Twelve patients have been treated using this modified triceps reflecting approach in the authors’ trauma center. Eight of these cases were previously reported as a part of a bicenter study including 24 patients, which demonstrated excellent quality of joint reconstruction and no relevant functional impairment (strength, Quick-DASH [Disabilities of Arm, Shoulder and Hand], Mayo Elbow Performance Score) [8]. We report on the three most recent cases, which were not included in the previous study.

**Fig. 10:**
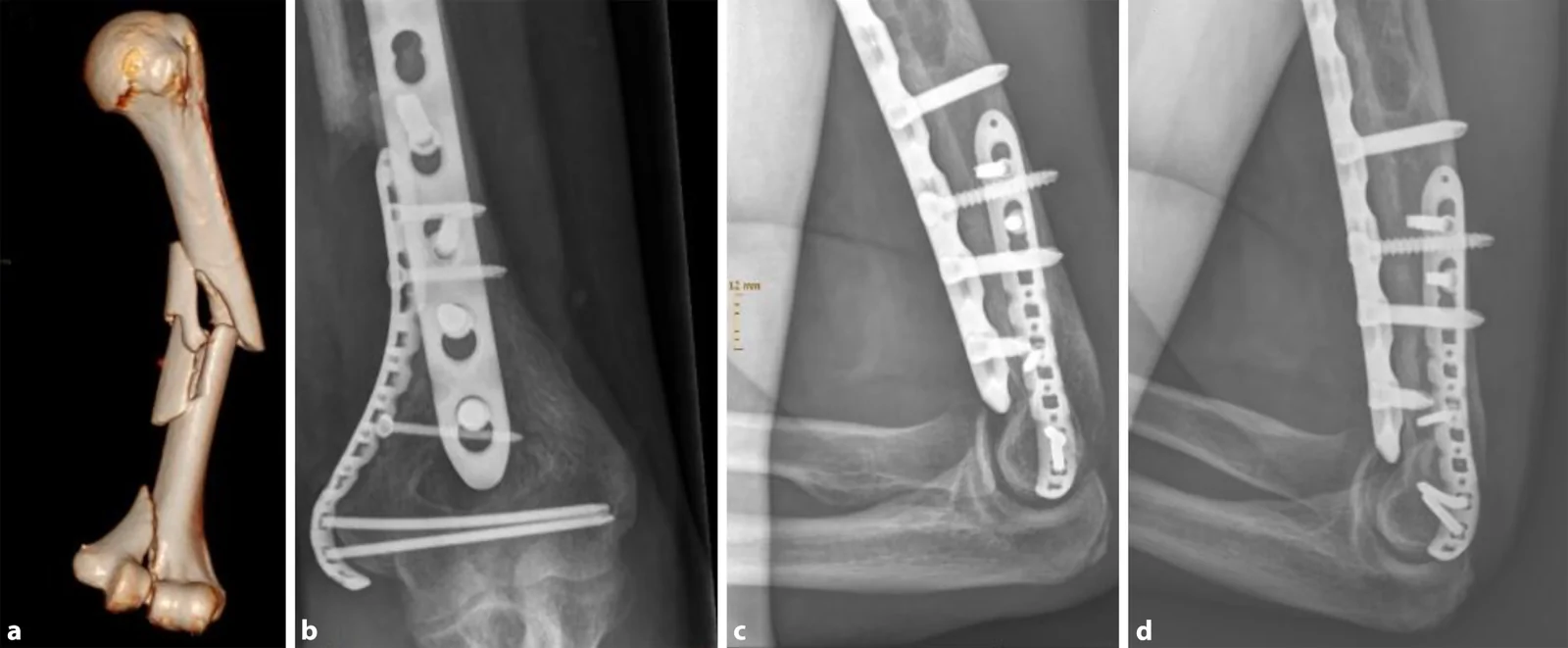
A 48-year-old man sustained a segmental humerus fracture with articular involvement. **a** Preoperative computed tomography. **b**, **c** X-rays obtained at the 3‑month follow-up with the flip osteotomy still visible. **d** After 1 year the flip osteotomy is fully healed

One patient was a 49-year-old man who sustained a grade 3 open segmental fracture of the left humerus with both a shaft fracture and distal intra-articular fracture (Fig. 10a) as well as rib fractures and a Lisfranc dislocation of the left foot after a traffic accident. The humeral shaft fracture was fixed with a plate via a minimally invasive anterolateral approach in supine position. The patient was then repositioned to prone to allow for a dorsal approach with a flip osteotomy to address the intra-articular distal humerus fracture. The remaining injuries were treated nonsurgically. The wounds healed uneventfully, and the patient was discharged home 8 days postoperatively. Twelve weeks after fracture fixation the patient displayed a range of motion of the elbow of flexion/extension 120/25/0° and did not report any pain or discomfort. The intra-articular fracture was no longer visible on the x‑rays; however the bone chip on the tip of the olecranon was still visible and not yet fully healed (Fig. 10b, c). At the 1‑year follow-up, the patient had almost achieved full range of motion with only a slight lack of extension of 15° remaining (flexion 140°, symmetrical to contralateral side).

**Fig. 11:**
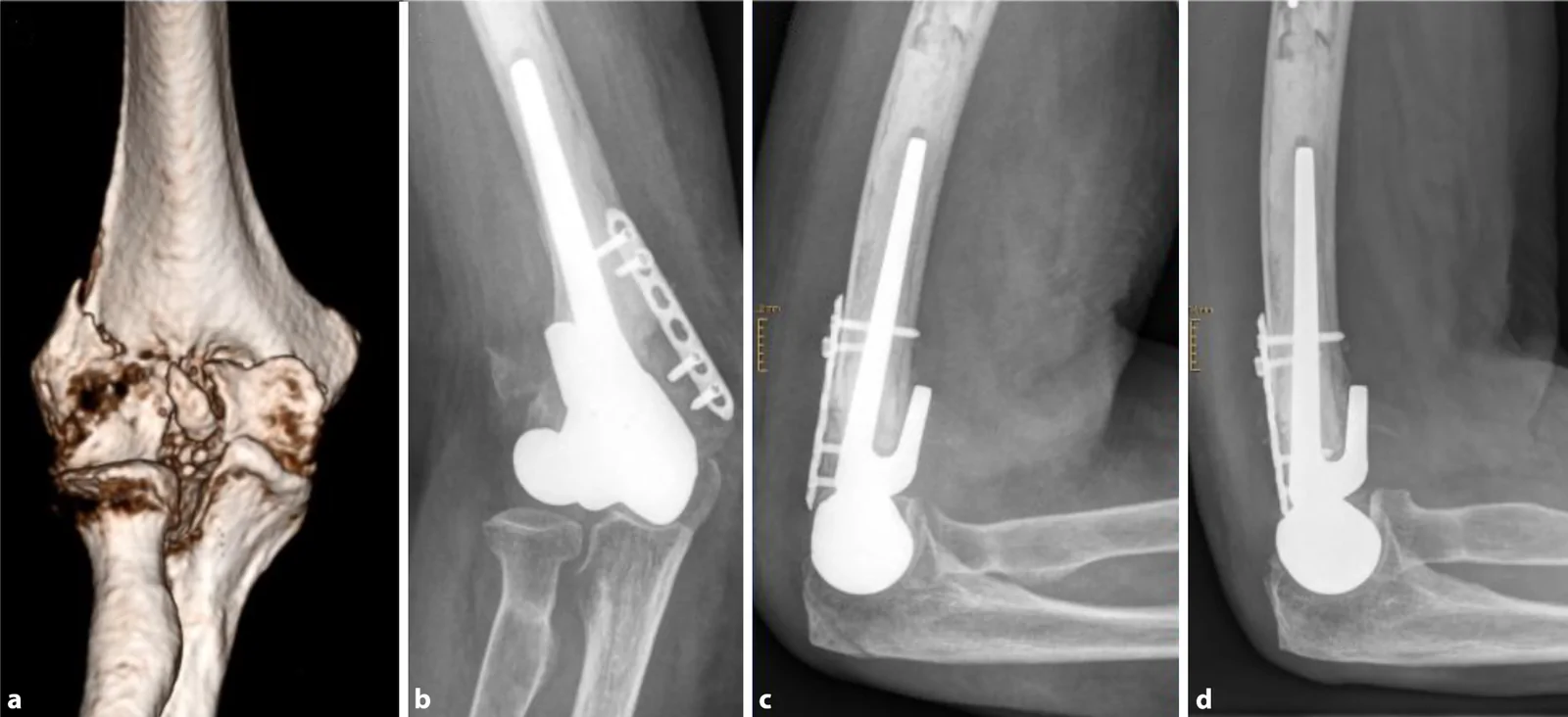
A 77-year-old woman sustained a distal intra-articular humerus fracture which was treated with a hemi-prosthesis and additional plate osteosynthesis. **a** Preoperative computed tomography. **b**, **c** X-rays obtained at the 3‑month follow-up. The flip osteotomy is still visible. **d** X-ray from the 6‑month follow-up showing the healed flip osteotomy

The second patient was a 77-year-old woman with known osteoporosis who sustained a distal intra-articular humerus fracture when she fell from standing height. The fracture was treated with a hemi-prothesis and an additional plate to fix the ulnar epicondyle. The prosthesis was implanted via a dorsal approach with flip osteotomy. The patient was discharged home 3 days postoperatively. The incision healed without any issues. At the 6‑month follow-up no complications had occurred, and the patient had achieved a range of motion of flexion/extension 110/20/0° and full range of pro-/supination. The x‑ray showed the flip osteotomy had fully healed (Fig. 11).

**Fig. 12:**
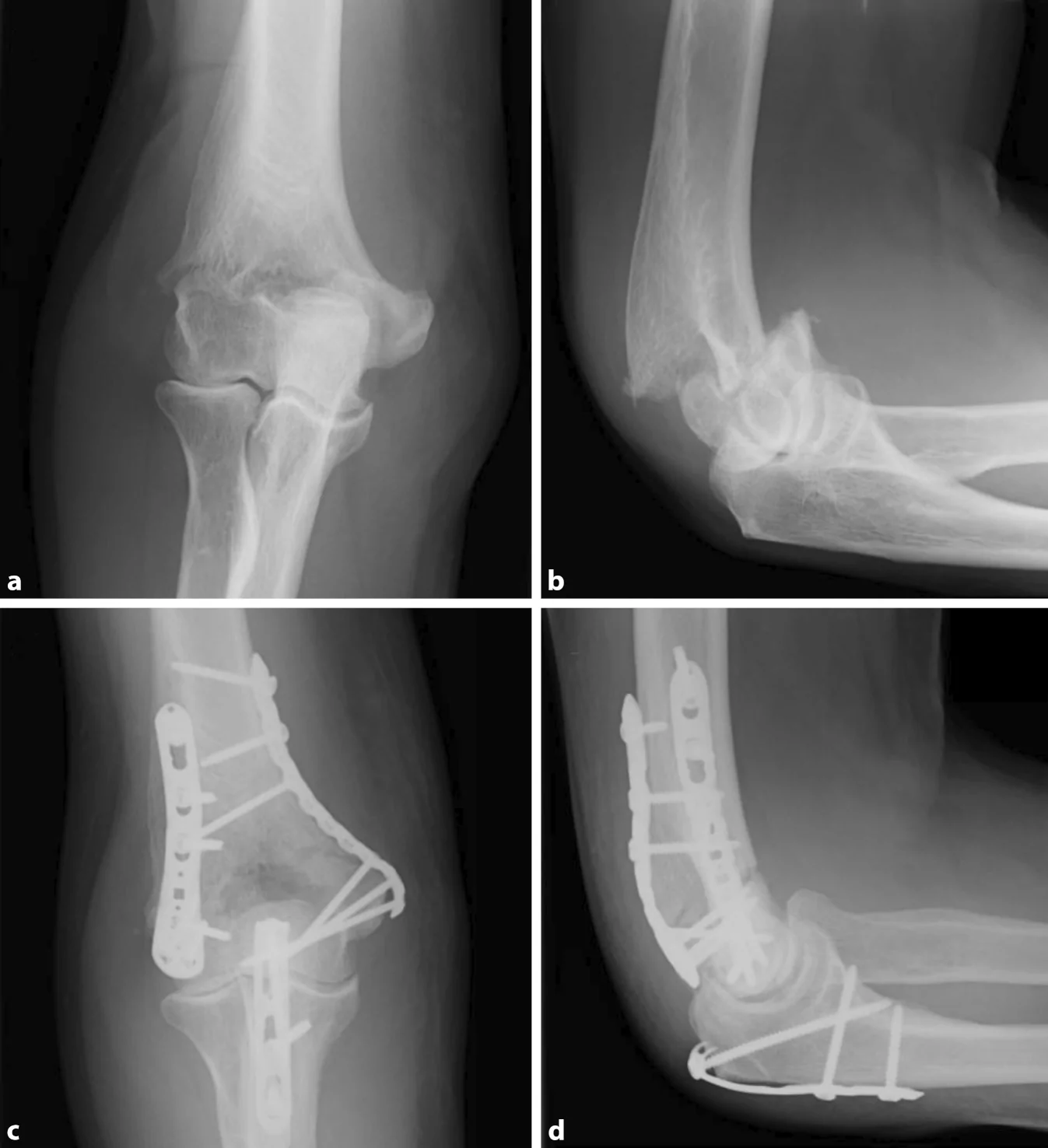
A 68-year-old man sustained an extra-articular fracture of the distal humerus treated with double plating using the flip osteotomy approach. The osteotomy was secured with a plate. **a**, **b** Preoperative images of the extra-articular fracture. **c**, **d** X-rays obtained at the 6‑week follow-up

The third patient was a 68-year old man who sustained a concussion injury and an extra-articular, transcondylar distal humerus fracture. The fracture was treated with two plates in a 90° configuration (Fig. 12). To enable good vision of this very distal fracture and facilitate anatomical reduction a flip osteotomy was performed. This was one of the first patients in which this approach was used by the authors. At this time a plate was used to fixate the olecranon chip. The authors no longer feel that plate fixation is warranted and recommend suture fixation as described above. Three months postoperatively the fracture had fully healed and at the 6‑month follow-up range of motion was flexion/extension 135/5/0° with full pro- and supination. However, the patient developed neuropathy of the ulnar nerve. The neuropathy was treated nonsurgically under guidance of both neurologists and plastic surgeons. One and a half years postoperatively the patient had regained feeling but continued to have a weakness in motor function.

## Data Availability

The data that support the findings of this study are available from the corresponding author upon reasonable request.

## Declarations

All patients included in the results section provided a general consent for the usage of their anonymous data for research purposes.
